# Stability of gene contributions and identification of outliers in multivariate analysis of microarray data

**DOI:** 10.1186/1471-2105-9-289

**Published:** 2008-06-20

**Authors:** Florent Baty, Daniel Jaeger, Frank Preiswerk, Martin M Schumacher, Martin H Brutsche

**Affiliations:** 1 Pulmonary Gene Research, University Hospital Basel, Petersgraben 4, Basel, Switzerland; 2 Department of Computer Science, University of Basel, Klingelbergstrasse 50, Basel, Switzerland; 3 Biomarker Development, Novartis AG, Klybeckstrasse 141, Basel, Switzerland

## Abstract

**Background:**

Multivariate ordination methods are powerful tools for the exploration of complex data structures present in microarray data. These methods have several advantages compared to common gene-by-gene approaches. However, due to their exploratory nature, multivariate ordination methods do not allow direct statistical testing of the stability of genes.

**Results:**

In this study, we developed a computationally efficient algorithm for: i) the assessment of the significance of gene contributions and ii) the identification of sample outliers in multivariate analysis of microarray data. The approach is based on the use of resampling methods including bootstrapping and jackknifing. A statistical package of R functions was developed. This package includes tools for both inferring the statistical significance of gene contributions and identifying outliers among samples.

**Conclusion:**

The methodology was successfully applied to three published data sets with varying levels of signal intensities. Its relevance was compared with alternative methods. Overall, it proved to be particularly effective for the evaluation of the stability of microarray data.

## Background

Ordination methods are useful tools for the analysis of gene expression microarrays. Principal component analysis (PCA) and correspondence analysis (CA) have both been used to extract the main sources of variation present in highly multivariate microarray data [1,2]. The supervised counterparts of these approaches, including between-group analysis (BGA) [3] and analyses with respect to instrumental variables [4], were proposed to handle descriptive variables controlled in the design of the experiment (e.g. disease classes). When dealing with transcriptomics data, multivariate approaches are generally more appropriate than univariate strategies because they intrinsically take gene covariations and interactions into account.

Constrained ordination methods are very efficient for sample classification and class prediction. They are flexible and can be used easily to identify groups of genes associated with classes of samples. Geometrical interpretations are generally required to investigate the gene-sample relationship. Genes of interest can also be ranked according to their discriminative power. However, considering the exploratory nature of these methods, it is not trivial to assess the significance of a given gene dysregulation in a multivariate setting. These methods rely on solving an eigenvalue problem whose solutions are given by the leading eigenvectors and whose theoretical statistical properties are particularly complex to study. To overcome this issue, resampling techniques have been proposed to estimate the stability of multivariate analyses. These techniques were described in a variety of scientific frameworks including environmetrics [5,6], chemometrics [7,8], and archaeology [9]. The general purpose is to develop inferential procedures for testing the statistical significance of the parameters provided by these exploratory techniques. Their applications are manifold, e.g. assessing which variables significantly contribute to the principal axes of a PCA, detecting outliers or influential observations. This approach has a great potential in the context of microarray data analysis as proposed by Tan and collaborators [10,11]. These authors described an application of bootstrapping to correspondence analysis. They outlined that their approach would have several advantages over classical gene-by-gene fits of ANOVA models. It particularly enables the extraction of lists of genes which are biologically more informative than those found by ANOVA.

In the present work, we propose a specific methodology for testing the stability of constrained ordination methods applied to microarray data. Unlike previous studies, our approach is dedicated to supervised multivariate analyses. To our knowledge, very few studies addressed the issue of stability assessment in supervised multivariate analyses. The potential of associating stability analysis in the supervised multidimensional context is multiple. By using the information of sample descriptors, genes can be associated with a given class of samples and the significance of this association can be assessed. A derived significance testing strategy regarding gene contributions is proposed. Further resampling methods based on jackknifing are also proposed to identify influential observations as an aid in outlier detection in microarray data sets. A comprehensive set of R functions illustrating our methodology was developed. The package is freely available on request.

The present manuscript is organized as follows. The first section introduces some theoretical aspects of ordination methods (with a particular focus on CA) and constrained ordination methods (especially BGA). The subsequent sections describe the different resampling strategies used in this project, as well as details about the algorithm. Illustrative examples demonstrating the implemented technique are given.

## Methods and Results

### Theory

#### Ordination methods

Both PCA and CA are commonly used in microarray data analysis. Some authors stressed that CA has several advantages over PCA [2,12]. Like other dimension reduction methods, CA summarizes structures in high-dimension space by projection onto a low dimension sub-space while loosing as little information as possible. Correspondence analysis involves a first step of symmetrical data transformation into a chi-square distance matrix which makes CA outputs particularly appropriate for the exploration of relationships between samples and genes. The mathematical basis of CA has been described elsewhere (see e.g. [13]) and will be briefly summarized. Thereafter observations are shown as rows and variables as columns.

Let us define the following:

• **Y**: the (*n *× *m*) matrix of gene expression data (*n *samples, *m *genes)

• **P **= **Y**/*N*: the data matrix divided by its grand total

• *r*: the *n*-dim vector of row sums of P

• *c*: the *m*-dim vector of column sums of P

• **D**_*r *_= diag(*r*): the diagonal matrix of row sums

• **D**_*c *_= diag(*c*): the diagonal matrix of column sums

The correspondence analysis of **Y **is obtained by carrying out the singular value decomposition of the doubled centered and standardized matrix **Z**:

(1) $$Z=D_{r}^{-1/2}(P-rc^{T})D_{c}^{-1/2}=U\Lambda V^{T}$$

with **Λ **the *k *× *k *(*k *= *rank*(**Z**)) diagonal matrix of singular values associated with **Z **with *λ*_1 _≥ ... ≥ *λ*_*k *_> 0, **U **an (*n *× *k*) matrix whose columns are the left singular vectors of **Z **and **V **an (*m *× *k*) matrix whose columns are the right singular vectors of **Z**. The rows of **U **and **V **are orthonormal with respect to **D**_*r *_and **D**_*c *_respectively:

(2) **U**^*T*^**D**_*r*_**U **= **V**^*T *^**D**_*c*_**V **= **I**

The principal components and row coordinates are respectively given by $D_{r}^{-1/2}$**U **and $D_{r}^{-1/2}$**UΛ**. The principal axes and column coordinates are respectively given by $D_{c}^{-1/2}$**V **and $D_{c}^{-1/2}$**VΛ**.

#### Constrained ordination methods

In microarray experiments, besides the main table **Y **containing the gene expression values, additional descriptive variables **X **controlled in the experimental design generally characterize samples. Constrained ordination methods aim to display the variations in the data which are explained by the descriptive variables. These two-table methods are dissymmetric because the information from **X **is used to constrain the analysis of **Y**. Correspondence analysis with respect to instrumental variables (CAIV) [4], which is closely related to PCA on instrumental variables [14], and between-group correspondence analysis [3] are two examples of constrained correspondence analysis which have been successfully applied to microarray data. In this field, these methods have been used for different purposes including sample classification [3], disease-class prediction [15], and removal of undesirable effects [4]. BGA is a particular case of CAIV when samples are characterized by one single categorical variable. Between-group correspondence analysis of table **Y **given the class descriptor **x **is simply the correspondence analysis of the table **Ŷ **corresponding to the table of means per group. BGA is the analysis of the per-class centroids. It provides up to *g *- 1 discriminating axes (*g *is the number of classes). The initial samples are thereafter projected as supplementary rows in the BGA sub-space. The BGA procedure provides the best linear combination of variables which maximizes the between-group variance.

#### Stability of gene contributions using bootstrapping

**Figure 1:**
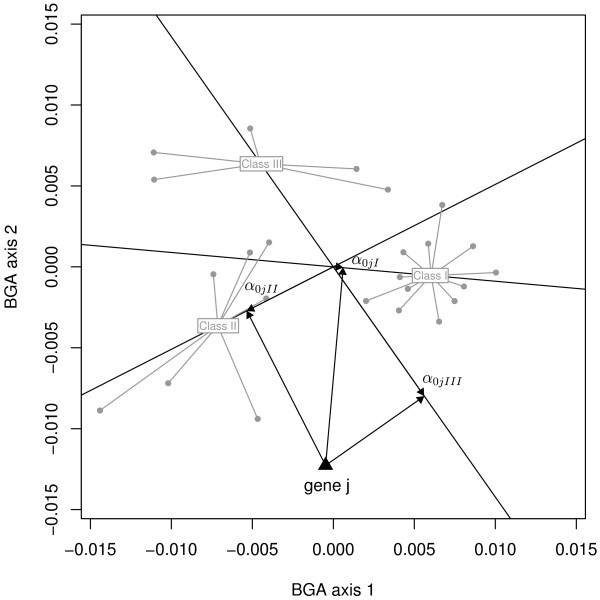
**Assessment of gene contributions by orthogonal projections**. The contribution of gene *j *toward the three classes of samples is measured by the distance *α*_0 _from the center of the BGA axes to the orthogonal projections onto the vectors of class centroids.

One interesting feature of BGA is the possibility to associate genes with pre-defined sample classes. The gene contribution towards sample classes is defined as the absolute distance from the center of the BGA axes to the orthogonal projections of the gene (*y*) onto the vector of the class centroid (*x*) (Figure 1). This distance is measured by the parameter: $\alpha_{0}=\frac{\langle x,y\rangle }{{\left\|x\right\|}^{2}}$. The higher |*α*_0_| is, the more important the gene contribution is. The sign of *α*_0 _indicates whether the gene is specifically up- or down-regulated, respectively for positive and negative values. Genes can be ranked according to their contribution towards each class modalities.

Introduced by Efron [16], bootstrapping is a distribution-free resampling method generally used to estimate the variance of estimators. Like other resampling techniques, bootstrapping provides a good alternative to establish the variability of an estimator. It is classically used to assess the bias and variance of model parameters, construct confidence intervals and rebuild empirical distributions. Several bootstrap refinements were implemented in the framework of techniques incorporating singular value decomposition [17,18]. In the present work, non-parametric bootstrapping was used to estimate the significance of gene contributions. The proposed bootstrapping strategy is based on the BGA model assumptions. As mentioned previously, the BGA of table **Y **with regard to the categorical variable **x**, is the analysis of the (*g *× *m*) table **Ŷ **corresponding to the per-class mean of **Y**. Let us define **X**, the (*n *× *g*) table of dummy variables coded from **x **and **Ŷ**_**x **_= **X**(**X**^*T*^**X**)^-1^**X**^*T*^**Y**, the (*n *× *m*) matrix of fitted values. Bootstrapped samples are built based on the residuals **E **= **Y **- **Ŷ**_**x**_. Residuals are sampled with replacement (E*) and new data sets are built as follow: Y* = **Ŷ**_**x **_+ E*.

The analysis of 100 to 1000 perturbated data sets is generally required to assess the parameters' distribution of a multivariate model (in our case, the gene contributions deduced from the gene and class-centroid coordinates). Out of these empirical distributions several indicators of the stability of gene contributions were calculated. Non-parametric 95% bootstrap confidence intervals were constructed using the 2.5% and 97.5% quantiles of the bootstrapped distribution. *z*-scores were defined for each gene as the ratio of the bootstrap estimates to its standard deviation. *p*-values were estimated according to the "bootstrapped eigenvector" procedure [6] as the probability of obtaining gene contributions equal to or smaller than zero for genes contributing positively in the original data set, or alternatively equal to or larger than zero for genes contributing negatively in the original data set.

Convex hulls were used to graphically display the spread of the bootstrapped gene coordinates on the dominant principal axes. The relative inertia of these gene coordinates was measured by the ratio of gene inertia to the total inertia.

Lebart [18] proposed two main categories of bootstrapping. Partial bootstrap makes use of *a posteriori *projections of the resampled elements on the original reference subspace provided by the analysis of the initial data set. Total bootstrap performs a new analysis on each of the resampled data sets. Both strategies have been implemented in the current project. It is noteworthy that partial bootstrap does not involve successive steps of singular value decomposition which makes it considerably faster than total bootstrap. Moreover, because total bootstrap requires a complete CA to be carried out for each bootstrapped table, the new sets of row and column coordinates belong to different subspaces which make their comparison more complex. Unlike partial bootstrap, total bootstrap is potentially subjected to axis reflection or inversion [19]. Signs of row and column coordinates in perturbated data sets can be inverted compared with the original data set. At least two approaches have been reported to overcome this drawback. The first consists in determining the sign of the correlations between the principal axes prior and after perturbations. A negative correlation indicates a reflexion which can be corrected by multiplying the new row and column coordinates by -1. Procrustes rotation was also proposed to fit the resampled row and column coordinates with the original scores and loadings [8,20,21]. The first (more conservative) option was implemented in the present work.

#### Detection of outliers using jackknifing

Jackknifing is another resampling technique introduced by Quenouille [22] and later developed by Tukey [23]. Jackknifed samples are built using a leave-one-sample-out strategy. Although jackknifing can be seen as a rough linear approximation of bootstrapping [16], it proves to be useful for investigating the influence of individual observations, as demonstrated for example in chemometrics by works from Martens and colleagues [7] or Westad and collaborators [8,21]). In the current paper, jackknifing was used to detect influential samples and outliers in microarray data sets. The number of resampled data sets created by jackknifing equals the number of samples in the original data set. Each new data set is identical to the initial data set except for one sample which is removed.

In a data set including *n *samples, *n *consecutive analyses are performed providing *n *sets of *n *- 1 sample coordinates. The impact of each individual sample on the other *n *- 1 samples is measured by the distance from the samples' original positions to their positions after resampling. If a given sample is highly influential, it may importantly impact the position of one or several other samples. A stability plot can be used to visualize the shift in the sample position after jackknifing. A large shift reflects the presence of an influential observation.

In order to identify observations which significantly influenced the position of other observations, the classical multivariate detection of outliers based on the Mahalanobis squared distance (*D*^2^) was used. These distances can be evaluated using a *χ*^2 ^distribution with the appropriate degrees of freedom. Each time a sample removal induced a shift to an extra sample $D_{i}^{2}>\chi_{0.975,p}^{2}$, the 0.975 quantile of a chi-square distribution, with *p *degrees of freedom, the sample was defined as significantly influential towards the extra sample. Overall, if the median of the *n *- 1 shifts induced by an observation is greater than a $\chi_{0.975,p}^{2}$ threshold, this observation was declared an outlier.

Similarly to total bootstrap, jackknife outcomes are potentially subjected to axis reflection. Sample coordinates were post multiplied by -1 in case of negative correlation between the principal components prior and after resampling.

#### Algorithm implementation

The implementation was done using R (with an extensive use of routines from the package ade4 [24]), and a new package *multistab *including original functions was developed. Our algorithm involves resampling techniques which are computationally intensive. The implementation allows the parallelization of the calculations. The R packages snow and Rmpi allow accessing the MPI/LAM subsystem, an implementation of the MPI standard, for distributing jobs among nodes. Calculations have been performed and tested on different configurations including computers with single and dual core CPUs as well as an MPI cluster of 16 heterogeneous nodes.

### Example of application

#### Data sets

Three publicly available data sets were used to illustrate the different features of our methodology. The first data set consists of a subset of data from the pioneer work of Bhattacharjee and colleagues [25] using gene expression profiling to investigate adenocarcinoma subclasses. The subset used in the current study includes 96 samples classified into 4 groups of patients (38 adenocarcinomas, 21 squamous cell carcinomas, 20 pulmonary carcinoids and 17 normal lung specimens). RNA extracts from tissue specimens (snap-frozen lung tumors and normal lung) were hybridized onto Affymetrix' hgu95a arrays. The second data set was published by Spira and colleagues [26] analyzing the airway epithelial cell transcriptome of smoking patients. In this study, 75 individuals classified into 3 groups (34 current smokers, 18 former smokers and 23 never smokers) were investigated. Bronchial cells were obtained from brushings of the right mainstem bronchus and RNA extracts were hybridized onto Affymetrix' hgu133a arrays. The third data set was described by Baty and colleagues [4] which investigated the effect of beverage consumption in healthy individuals. One hundred and eight samples classified in 5 groups (21 baseline, 20 alcohol, 22 grape juice, 23 water and 22 wine) were analyzed. RNA extracts from peripheral blood leukocytes were hybridized onto Affymetrix' hgu133a arrays. The transcriptomics signal is expected to be high in Bhattacharjee (tumor cells from well-defined lung cancer patients), intermediate in Spira (mixture of bronchial cells in a population of smokers), and low in Baty (physiologic variations of blood cells in healthy patients).

##### Significance of gene contributions

**Figure 2:**
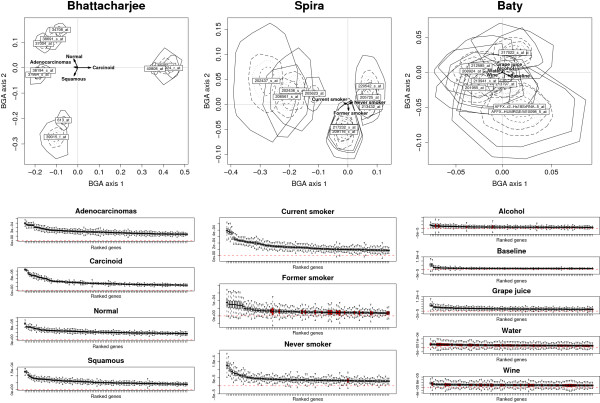
**Stability of gene contributions using bootstrapping**. Uncertainty plots in the upper panels display for each data set the coordinates of the 10 most discriminating genes after partial bootstrap (500 repetitions) in the first two axes of BGA. Convex hulls containing 25%, 50%, 75% and 100% of the points are used to represent the spread of gene coordinates. The directions of class centroids are represented by arrows. In the lower panels, sensitivity boxplots show the distributions of gene contributions. Genes are ranked from left to right according to their discriminating power. The zero threshold is depicted as a dashed line. Gene distributions where more than 5% of values are below 0, are represented as plain boxplots.

Five hundred bootstrapped samples were built in order to assess the empirical distribution of the gene contributions within each data set. Partial bootstrap was used and the newly calculated gene coordinates were represented with convex hulls on the factorial map defined by the two first principal axes of the BGA (Figure 2, upper panels). The size of the convex hulls, measured in terms of relative inertia, gives indications about the gene stability. The discriminative power of genes is measured by the distance from the center of the BGA axes to the hull locations. Only the 10 most discriminating genes for each data set are displayed. The degree of hull overlap for the different combinations of factor levels, gives important indication about the specificity of the gene discrimination. The boxplots of gene contributions (Figure 2, lower panels) was used to estimate the proportion of overly unstable genes (genes with a *p*-value ≥ 0.05). The proportion of overly unstable genes is refered to as false positive rate.

Gene hulls in Bhattacharjee were small (the average relative inertia of the 100 most discriminating genes was 0.01), distant from the center of the plot and the group of genes associated with each factor level did not overlap. This documents the stability and specificity of the markers found in this experiment. This result was further confirmed by the boxplot representation showing the distributions of gene contributions (Figure 2, lower pannels). In Bhattacharjee, all most discriminating genes significantly contributed to the class discrimination since no distribution crossed the 0 threshold (*p *< 0.002). Thus, the false positive rate was 0%.

On the other hand, in the data sets of Spira and Baty, the relative inertia of gene hulls was larger (the average relative inertia was 0.06 and 0.11 respectively). In the data set of Baty, the degree of hull overlapping was particularly high. The level of false positive rate was moderate in Spira data (8%) and high in Baty data (32%). This level differed from one experimental condition to one another. In Spira, the "Former smoker" group had a higher false positives rate (21%) compared to "Current smoker" (0%) and "Never smoker" (1%). The "Current smoker" category was the one with the most stable gene signals. In Baty, the false positive rate was high. Genes associated with the consumption of water were highly unstable (false positive rate of 70%). Overall the false positive rate was measured for the 100 genes with the highest ranking in terms of gene contribution. As to be expected, this rate increased with lower gene ranks.

##### Identification of influential observations and outliers

**Figure 3:**
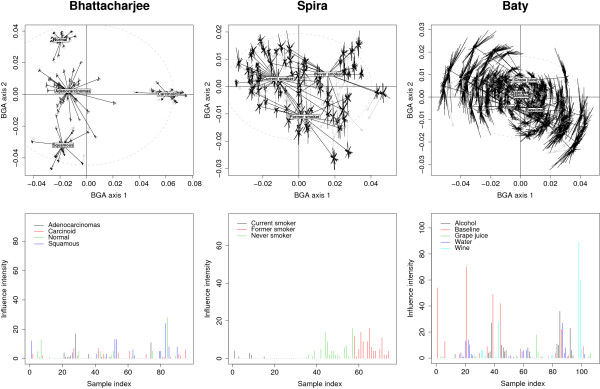
**Detection of influential observations and outliers by jackknifing**. Stability plots in the upper panels show the shifts of sample coordinates induced by jackknifing in the first two axes of BGA. The dashed ellipse delineate 2 standard deviations of the sample coordinates on the displayed axes. Barplots in the lower panels show how many times samples were declared as significantly influential.

Jackknifing was used to estimate the influence of each observation on the sample coordinates. The stability plots presented in the upper panels of Figure 3 display the sample coordinates (grouped by class) prior and after resampling in the two dominant axes of BGA. The *n *- 1 positions of each sample were represented with star plots showing the shifts induced by sample removals. In Bhattacharjee data, the relative shifts induced by jackknifing were small (Figure 3, upper left panel). These shifts were much more prominent in Spira and Baty data sets. The barplots in Figure 3 (lower panels) display graphically the degree of influence of each sample. For example, the first sample in Bhattacharjee was found to influence significantly the position of 12 different samples. By comparing the median shift induced by each single sample with a $\chi_{0.975,p}^{2}$ threshold, no sample was declared an outlier in both Bhattacharjee and Spira data sets, whereas 6 samples were found to be outliers in Baty. In Spira, one can easily observe that the influential observations are mostly identified in the patient groups "Former smoker" and "Never smoker". The ouliers in Baty belonged to the "Baseline" and "Wine" group.

#### Comparison with alternative approaches

The relevance of the results obtained by bootstrapped BGA was compared with those obtained by bootstrapped correspondence analysis [11] and the gene-by-gene fit of 1-way ANOVA models. The 'sarcoidosis' data set provided in our R package was used in this comparative study. This data set, which was previously described [15,27], included 24 individuals (12 healthy controls vs. 12 sarcoidosis patients). The group of sarcoidosis patients was subdivided into 7 sarcoidosis stage I (low severity) and 5 sarcoidosis stage ≥ II (higher severity) patients. Messenger RNA extracted from peripheral blood were hybridized on Affymetrix' hgu95a arrays. The data were normalized using the 'vsn' algorithm. After gene filtration, 7206 genes remained in the data set. Bootstrapped BGA was performed using 1000 iterations and 84 highly significant genes (*p *< 0.001) among the genes which mostly participate to the between group discrimination were selected. Hundred and twelve genes with the highest contribution on the first axis in bootstrapped CA were selected. A third list of genes included the 100 most significant genes obtained by ANOVA models. There were 18 genes overlapping between the list from bootstrapped CA and bootstrapped BGA, 27 genes beween bootstrapped BGA and ANOVA and 7 between bootstrapped CA and ANOVA. A functional analysis was carried out on these 3 lists of genes using the web tool DAVID [28]. Table 1 summarizes the 11 significantly enriched Gene Ontology (GO) categories found from the list of genes obtained in bootstrapped BGA. The number of GO categories identified before adjustment for *p*-values was slightly more diverse in bootstrapped BGA (*n *= 70) than in bootstrapped CA or ANOVA (respectively *n *= 52 and *n *= 55). On the other hand, the enrichment of biologically informative genes in each GO categories was higher in bootstrapped BGA than in bootstrapped CA and ANOVA. This is in agreement with previously reported findings [4,11].

**Table 1:** Functional analysis of genes obtained by bootstrapped BGA, bootstrapped CA and ANOVA

Functional category of genes	Benjamini-Hochberg adjusted *p*-values		
	Bootstrapped BGA	Bootstrapped CA	ANOVA
Response to stress	24% (p = 0.01)	18% (*p *= 0.04)	17% (*p *= 0.76)
Defense response	25% (*p *< 0.01)	16% (*p *= 0.38)	15% (*p *= 0.85)
Immune response	24% (*p *< 0.01)	15% (*p *= 0.39)	13% (*p *= 0.95)
Humoral immune response	11% (*p *< 0.01)	4% (*p *= 0.99)	5% (*p *= 0.85)
Response to biotic stimulus	25% (*p *< 0.01)	16% (*p *= 0.48)	16% (*p *= 0.79)
Response to stimulus	37% (*p *= 0.01)	24% (*p *= 0.62)	26% (*p *= 0.79)
Response to pest, pathogen or parasite	16% (*p *= 0.01)	12% (*p *= 0.09)	11% (*p *= 0.98)
Response to other organism	16% (*p *= 0.02)	12% (*p *= 0.10)	11% (*p *= 0.95)
Gas transport	5% (*p *= 0.03)	0% (NS)	3% (*p *= 0.82)
Oxygen transport	5% (*p *= 0.03)	0% (NS)	3% (*p *= 0.82)
Humoral defense mechanism	8% (*p *= 0.05)	0% (NS)	5% (*p *= 0.97)

The Gene Ontology analysis is based on the 11 most significant GO categories (adjusted *p*-values < 0.05) obtained after bootstrapped BGA. The results present the proportion of genes that belong to these GO categories (%) and the enrichment significance (*p*-values are adjusted using the Benjamini-Hochberg method [30]), after bootstrapped BGA, bootstrapped CA and ANOVA.

The advantage of using multivariate versus univariate approaches in highly multivariate data such as microarray data has been already well documented in the literature. The extraction of meaningful gene mechanisms implies that genes are treated as a whole and not separately. This explains why higher enrichment of biologically meaningful GO categories (or functional pathways) are generally obtained when using multivariate approaches compared with univariate approaches. The choice of using unsupervised or supervised ordination methods mainly depends on the objectives of the study. When the biologist is interested in finding groups of discriminating genes that explain differences among well-defined patients categories, BGA is a method which should be considered. Because the grouping information is directly incorporated in the BGA model, the dimension reduction of the multivariate data is driven by the phenotypic information. This generally provides noticeable simplification of data interpretation. In contrast, when using unconstrained CA, one first extracts the major compositional variation present in the leading axes then relates this variation to external information. In certain situations, the leading axes are subject to unexpected sources of variation, making their interpretation difficult.

When the design of experiment tends to become more complex, (e.g. when several controlled variables are included), other multivariate approaches can be used to incorporate this external information into a constrained CA model (see e.g. [4]). Double constrained CA can also be suitable if one wants to incorporate external information on both rows and columns [29]. However, some more complex models of constrained CA including interactions and contrasts might be difficult to interpret. When the number of constraints tends to be too large, a phenomenon of relaxation of constraints may happen. In such cases, simple unconstrained CA might appear appropriate.

## Discussion

The bootstrapping technique presented in this study proved to be very useful to evaluate gene stability in microarray data. The three data sets were chosen with an *a priori *knowledge about the strength of their transcriptomics signal. The biologically most well-defined data set was Bhattacharjee with four clearly distinct categories of patients and samples taken from distinct types of lung tumors. The class discrimination was high (proportion of explained inertia = 38%; *p *< 0.001) and the gene signal very stable and specific. A less stable signal came out of Spira data, where samples were derived from a mixture of airway epithelial cells and the patient groups less clear cut. The identification of stable signals specific to "Former smoker" was particularly difficult although the between-class discrimination was significant (proportion of explained variance = 6%; *p *= 0.006). The weakest and highly unstable signals were found in Baty data, where the expected effect was within the physiological range of normal cells. The proportion of explained variance was very low in this example (4%; *p *= 0.46).

Jackknifing was particularly efficient to detect influential observations or outliers in our setting. This method provided important diagnostic insights in the data as well as the experimental design. A careful exploration of the sample stability can help the experimenter to identify samples with imprecise or wrong group allocation, or group with a heterogeneous behaviour. Furthermore, this method can be used to identify poorly specified sample categories or subcategories of samples. As an example, further investigations might reveal that the "Former smoker" category in Spira might share gene signatures both from "Never smoker" and "Current smoker". With the proposed tools, researchers can identify inconsistent observations/samples or groups and have a strategy at hand to correct for imprecise descriptions in case of sufficient respective evidence.

Particular attention was paid to the computational aspects of the resampling calculations. The calculations were considerably accelerated by the use of parallelization. Furthermore, routines used to carry out CA and BGA (originally proposed in the R package ade4) have been optimized for the analysis of gene expression data where the number of variables far exceeds the number of samples. Performance testing on data sets of different size showed an improvement of calculation time by a factor 10 to 50. As previously mentioned, the method of partial bootstrap was prefered to total bootstrap for testing the stability of gene contributions since it was simpler to perform and computationally more efficient (approximately twice as fast).

## Conclusion

Dimension reduction methods are powerful tools that help biologists exploring their data and generate new hypotheses. Like other supervised approaches, constrained ordination methods incorporate external information that greatly simplifies the interpretation of microarray data analysis. The principal axes of BGA being defined as the linear combination of genes that maximizes the between-group variance, it is straightforward to extract groups of genes that discriminate between disease categories. By using the resampling methodology described above, it is possible to assess the reliability of solutions in a multivariate analysis of gene expression microarray data. Although both bootstrapping and jackknifing should not be used for formal statistical hypothesis testing, they proved to be useful to identify highly consistent genes, filter out some false positive genes, and to allow detection of influential observations among samples. With regard to this complementary information, the biologist can decide to pay more attention to highly stable discriminating genes, which in turn can be used for subsequent formal statistical hypothesis testing. Based on jackknifing information, the biologist can also decide on croping outlying observations or refining *a priori *sample classification.

In conclusion, the methodology and the collection of tools proposed in this study are suitable for the assessment of the significance of gene contributions and the detection of outliers in microarray data and this in a multivariate fashion. The set of R functions includes additional functions which test the stability of multivariate analysis results. Overall, the R package we developed constitutes a novel and comprehensive suite of diagnostic tools to evaluate the robustness of multivariate representations of high-throughput gene expression data.

## Authors' contributions

FB developed the algorithm, performed the analysis and wrote the paper. DJ and FP contributed to the implementation and the optimization of the algorithm. MMS contributed in the development of the algorithm. MHB supervised the work and was significantly involved in the writing of the manuscript. All co-authors read and approved the manuscript.
